# Crystal structure of 2,2′-(ethane-1,2-di­yl)bis­(2,3-di­hydro-1*H*-naphtho­[1,2-*e*][1,3]oxazine): a prospective raw material for polybenzoxazines

**DOI:** 10.1107/S2056989017006673

**Published:** 2017-05-09

**Authors:** Augusto Rivera, Juan E. Cepeda-Santamaría, Jaime Ríos-Motta, Michael Bolte

**Affiliations:** a Universidad Nacional de Colombia, Sede Bogotá, Facultad de Ciencias, Departamento de Química, Cra 30 No. 45-03, Bogotá, Código Postal 111321, Colombia; b Institut für Anorganische Chemie, J. W. Goethe-Universität Frankfurt, Max-von Laue-Strasse 7, 60438 Frankfurt/Main, Germany

**Keywords:** crystal structure, short contacts, benzoxazines, phenolic resins

## Abstract

In the title compound, C_26_H_24_N_2_O_2_, the oxazine moiety is fused to a naphthalene ring system. The asymmetric unit consists of one half of the mol­ecule, which lies about an inversion centre. The C atoms of the ethyl­ene spacer group adopt an anti­periplanar arrangement. The oxazine ring adopts a half-chair conformation. In the crystal, supra­molecular chains running along the *b* axis are formed *via* short C—H⋯π contacts. The crystal studied was a non-merohedral twin with a fractional contribution of 0.168 (2) of the minor twin component.

## Abstract 2

The mol­ecular and crystal structures of the centrosymmetric naphthoxazine derivative is reported. In the absence of hydrogen-bonding and π–π stacking inter­actions, the crystal structure is stabilized by short C—H⋯π contacts.

## Chemical context   

The oxazine moiety is well known as a building block for high-performance phenolic resins, which are of great inter­est in industry due to their superior mechanical and physical properties together with unusually high thermal resistance (Kiskan & Yagci, 2005 ▸). Recently, because of their high flexibility in mol­ecular design and performance-to-cost ratio, these monomers have gained attention for the preparation of cured thermosetting resins (Song *et al.*, 2014 ▸; Yeganeh & Jangi, 2010 ▸). Benzoxazines and naphthoxazines, originally proposed by Holly & Cope (1944 ▸) and subsequently elaborated by Burke and co-workers (Burke *et al.*, 1952 ▸), are obtained by Mannich-type condensation–cyclization reactions of phenols or naphthols with formaldehyde and primary amines in a 1:2:1 ratio (Deck *et al.*, 2014 ▸). Inter­est in the synthesis of polybenzoxazines and polynaphthoxazines has greatly increased during the past few years as they have a great deal of mol­ecular design flexibility compared to ordinary phenolics (Yildirim *et al.*, 2006 ▸). The title bis­napthoxazine, 2,2′-(ethane-1,2-di­yl)bis­(2,3-di­hydro-1*H*-naphtho­[1,2-*e*][1,3]oxazine), C_26_H_24_N_2_O_2_, was prepared by condensation of 2-naphthol with ethyl­enedi­amine and formaldehyde in a 2:1:4 molar ratio at room temperature for 15 min in methanol solution. Evaporation at room temperature afforded the title compound in 73% yield after recrystallization.
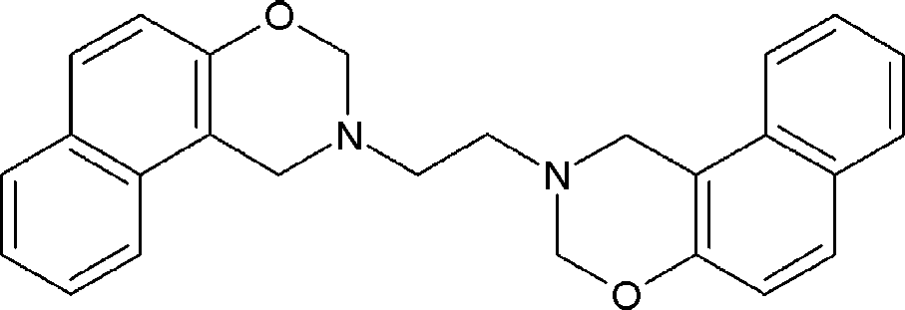


## Structural commentary   

**Figure 1:**
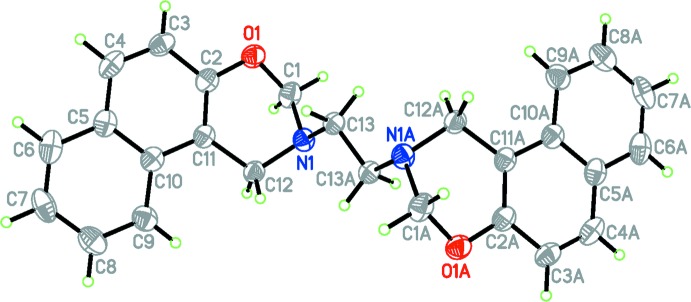
The mol­ecular structure of the title compound, with displacement ellipsoids drawn at the 50% probability level. Atoms labelled with the suffix A are generated using the symmetry operator (1 − *x*, 1 − *y*, 1 − *z*).

In general terms, the structure of the title compound (Fig. 1 ▸) is similar to those of other naphthoxazine derivatives that have been reported in that the oxazine moiety adopts a half-chair conformation (Yang *et al.*, 2007 ▸; Rivera *et al.*, 2015 ▸), with puckering parameters *Q* = 0.478 (3) Å, θ = 51.5 (4)°, φ = 86.6 (4)°, and the ethyl­ene spacer group adopts an anti­periplanar arrangement as observed in 3,3′-(ethane-1,2-di­yl)bis­(3,4-di­hydro-2*H*-1,3-benzoxazine) (Rivera *et al.*, 2012 ▸), with a N1—C13—C13^i^—N1^i^ torsion angle of 180.0° [symmetry code: (i) 1 − *x*, 1 − *y*, 1 − *z*]. However, unlike the related structures, which crystallized in monoclinic space groups with one mol­ecule in the asymmetric unit (Yang *et al.*, 2007 ▸; Rivera *et al.*, 2012 ▸, 2015 ▸), the title compound (I) crystallizes with just half a mol­ecule in the asymmetric unit in the space group *P*2_1_/*c*, utilizing the crystallographic inversion centre in the mol­ecular symmetry. The other half of the mol­ecule is generated by the symmetry operation (1 − *x*, 1 − *y*, 1 − *z*).

The aromatic C—C bonds of naphthalene ring system have a narrow range of distances [from 1.365 (5) to 1.431 (4) Å]. The central C5—C10 bond at 1.415 (4) Å is, however, shorter by 0.014 Å than those in related structures (Yang *et al.*, 2007 ▸; Rivera *et al.*, 2015 ▸). The N1—C1 and O1—C1 bond lengths are normal and comparable to the corresponding values observed in these related structures.

## Supra­molecular features   

**Figure 2:**
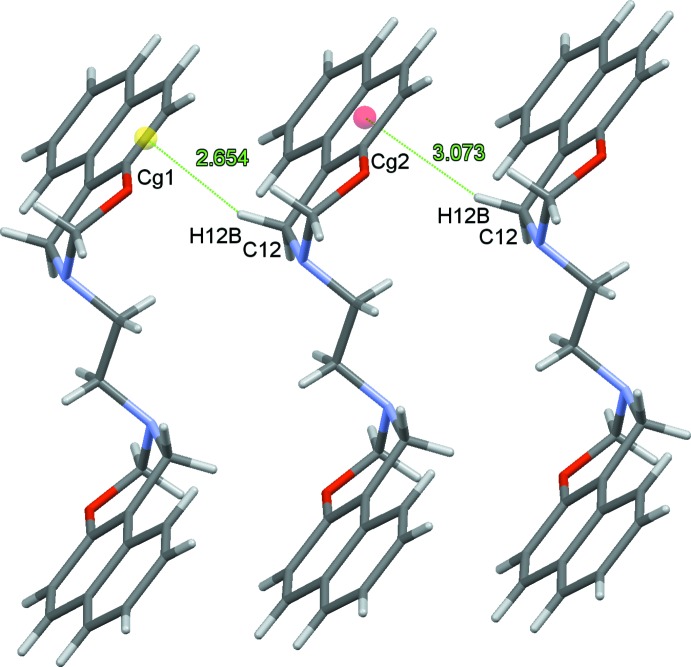
Possible C—H⋯π contacts, shown as dotted green lines, between mol­ecules of (I). Bond mid-points and ring centroids are shown as colored spheres.

**Figure 3:**
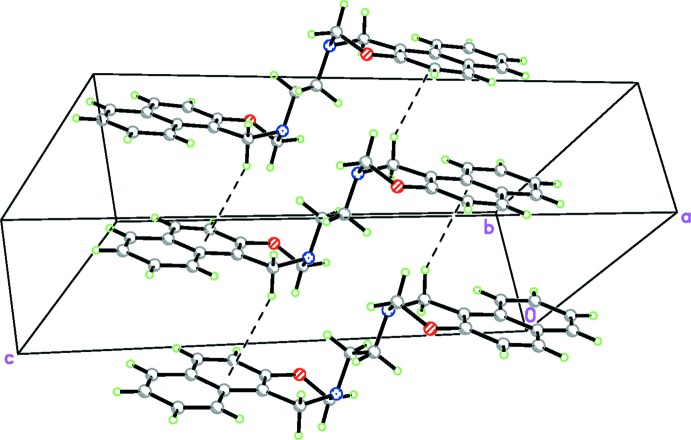
Crystal packing of (I), showing C—H⋯(C,C) short contacts that result in chains propagating along the *b-*axis direction.

In the crystal, the packing of the title compound is dominated by short contacts (Table 1 ▸), as indicated by a *PLATON* (Spek, 2009 ▸) analysis. These contacts result from short C12—H12*B*⋯C2 and C12—H12*B*⋯C3 separations, which at 2.75 Å are both 0.15 Å shorter than the sum of the van der Waals radii, while the C—H⋯*Cg*1 contact to the mid-point of the C2–*-*C3 bond is even shorter at approximately 2.65 Å. These contacts are also much shorter than the C—H⋯*Cg*2 contact to the centroid of the C2–C4/C11/C12 ring (Fig. 2 ▸). The mol­ecules are by these short C—H⋯π contacts linked into chains propagating along the *b*-axis direction (Fig. 3 ▸).

## Database survey   

The title compound is the first example of two naphtho-oxazine moieties linked by an ethyl­ene bridge.

## Synthesis and crystallization   

The title compound was prepared as described by Rivera *et al.* (2006 ▸). Crystals were obtained by slow evaporation of the reaction solution at ambient temperature and were isolated from the solution before complete evaporation of the solvent mixture.

## Refinement details   

Crystal data, data collection and structure refinement details are summarized in Table 2 ▸. All H atoms were located in the difference electron-density map. C-bound H atoms were fixed geometrically (C—H = 0.95 or 0.99 Å) and refined using a riding-model approximation, with *U*_iso_(H) set to 1.2*U*_eq_ of the parent atom. The crystal was a non-merohedral twin with a fractional contribution of 0.168 (2) of the minor twin component.

## Supplementary Material

Crystal structure: contains datablock(s) I. DOI: 10.1107/S2056989017006673/sj5529sup1.cif

Structure factors: contains datablock(s) I. DOI: 10.1107/S2056989017006673/sj5529Isup2.hkl

Supporting information file. DOI: 10.1107/S2056989017006673/sj5529Isup3.cml

CCDC reference: 1547729

Additional supporting information:  crystallographic information; 3D view; checkCIF report

## Figures and Tables

**Table 1:** Selected short-contact geometry (Å, °) *Cg*1 is the mid-point of the C2—C3 bond and *Cg*2 is the centroid of the C2–C4/C11/C12 ring.

C—H⋯C	H⋯C	C—H⋯C
C12—H12*B*⋯C2^i^	2.75	169
C12—H12*B*⋯C3^i^	2.75	142
C12–H12*B*⋯*Cg*1	2.654	157
C12–H12*B*⋯*Cg*2	3.073	155

Symmetry code: (i) *x*, −1 + *y*, *z*.

**Table 2:** Experimental details

Crystal data	
Chemical formula	C_26_H_24_N_2_O_2_
*M*_r_	396.47
Crystal system, space group	Monoclinic, *P*2_1_/*c*
Temperature (K)	173
*a*, *b*, *c* (Å)	9.8658 (10), 5.0979 (4), 19.551 (2)
β (°)	96.033 (8)
*V* (Å^3^)	977.87 (16)
*Z*	2
Radiation type	Mo *K*α
μ (mm^−1^)	0.09
Crystal size (mm)	0.27 × 0.11 × 0.04

Data collection	
Diffractometer	Stoe IPDS II two-circle
Absorption correction	Multi-scan (*X-AREA*; Stoe & Cie, 2001 ▸)
*T*_min_, *T*_max_	0.443, 1.000
No. of measured, independent and observed [*I* > 2σ(*I*)] reflections	9335, 9335, 5706
(sin θ/λ)_max_ (Å^−1^)	0.625

Refinement	
*R*[*F* ^2^ > 2σ(*F* ^2^)], *wR*(*F* ^2^), *S*	0.063, 0.130, 0.94
No. of reflections	9335
No. of parameters	137
H-atom treatment	H-atom parameters constrained
Δρ_max_, Δρ_min_ (e Å^−3^)	0.53, −0.34

Computer programs: *X-AREA* (Stoe & Cie, 2001 ▸), *SHELXT* (Sheldrick, 2015*a* ▸), *XP* in *SHELXTL-Plus* (Sheldrick, 2008 ▸), *SHELXL2016* (Sheldrick, 2015*b* ▸) and *publCIF* (Westrip, 2010 ▸).

## supplementary crystallographic information

### Crystal data

**Table:**

C_26_H_24_N_2_O_2_	*F*(000) = 420
*M**_r_* = 396.47	*D*_x_ = 1.347 Mg m^−^^3^
Monoclinic, *P*2_1_/*c*	Mo *K*α radiation, λ = 0.71073 Å
*a* = 9.8658 (10) Å	Cell parameters from 9056 reflections
*b* = 5.0979 (4) Å	θ = 2.8–26.4°
*c* = 19.551 (2) Å	µ = 0.09 mm^−^^1^
β = 96.033 (8)°	*T* = 173 K
*V* = 977.87 (16) Å^3^	Needle, colourless
*Z* = 2	0.27 × 0.11 × 0.04 mm

### Data collection

**Table:**

Stoe IPDS II two-circle diffractometer	9335 independent reflections
Radiation source: Genix 3D IµS microfocus X-ray source	5706 reflections with *I* > 2σ(*I*)
ω scans	θ_max_ = 26.4°, θ_min_ = 2.8°
Absorption correction: multi-scan (X-AREA; Stoe & Cie, 2001)	*h* = −12→12
*T*_min_ = 0.443, *T*_max_ = 1.000	*k* = −6→6
9335 measured reflections	*l* = −24→24

### Refinement

**Table:**

Refinement on *F*^2^	0 restraints
Least-squares matrix: full	Hydrogen site location: inferred from neighbouring sites
*R*[*F*^2^ > 2σ(*F*^2^)] = 0.063	H-atom parameters constrained
*wR*(*F*^2^) = 0.130	*w* = 1/[σ^2^(*F*_o_^2^) + (0.050*P*)^2^] where *P* = (*F*_o_^2^ + 2*F*_c_^2^)/3
*S* = 0.94	(Δ/σ)_max_ < 0.001
9335 reflections	Δρ_max_ = 0.53 e Å^−^^3^
137 parameters	Δρ_min_ = −0.34 e Å^−^^3^

### Special details

**Table:**

Geometry. All esds (except the esd in the dihedral angle between two l.s. planes) are estimated using the full covariance matrix. The cell esds are taken into account individually in the estimation of esds in distances, angles and torsion angles; correlations between esds in cell parameters are only used when they are defined by crystal symmetry. An approximate (isotropic) treatment of cell esds is used for estimating esds involving l.s. planes.
Refinement. Refined as a 2-component twin

### Fractional atomic coordinates and isotropic or equivalent isotropic displacement parameters (Å^2^)

**Table:**

	*x*	*y*	*z*	*U*_iso_*/*U*_eq_	
N1	0.3328 (2)	0.3467 (5)	0.51587 (12)	0.0291 (6)	
O1	0.14340 (19)	0.6404 (5)	0.52587 (10)	0.0346 (5)	
C1	0.1935 (3)	0.4104 (7)	0.49256 (16)	0.0351 (8)	
H1A	0.135064	0.258586	0.501152	0.042*	
H1B	0.185528	0.440900	0.442290	0.042*	
C2	0.1675 (3)	0.6281 (6)	0.59661 (15)	0.0302 (7)	
C3	0.0926 (3)	0.8089 (6)	0.63272 (17)	0.0350 (8)	
H3	0.029986	0.925542	0.608217	0.042*	
C4	0.1107 (3)	0.8150 (7)	0.70288 (17)	0.0370 (8)	
H4	0.061911	0.939265	0.726968	0.044*	
C5	0.2015 (3)	0.6382 (7)	0.74028 (15)	0.0319 (7)	
C6	0.2182 (3)	0.6366 (7)	0.81341 (16)	0.0397 (8)	
H6	0.169242	0.759116	0.837964	0.048*	
C7	0.3039 (3)	0.4611 (7)	0.84895 (16)	0.0419 (9)	
H7	0.313599	0.460117	0.897805	0.050*	
C8	0.3771 (3)	0.2834 (7)	0.81263 (17)	0.0421 (9)	
H8	0.436829	0.162438	0.837424	0.051*	
C9	0.3648 (3)	0.2792 (7)	0.74225 (15)	0.0355 (8)	
H9	0.415732	0.155997	0.718946	0.043*	
C10	0.2761 (3)	0.4585 (6)	0.70373 (15)	0.0295 (7)	
C11	0.2593 (3)	0.4567 (6)	0.63016 (15)	0.0276 (7)	
C12	0.3413 (3)	0.2746 (6)	0.58900 (14)	0.0288 (7)	
H12A	0.437938	0.278593	0.608671	0.035*	
H12B	0.307562	0.092959	0.593092	0.035*	
C13	0.4289 (2)	0.5552 (6)	0.50126 (15)	0.0290 (7)	
H13A	0.430945	0.691415	0.537435	0.035*	
H13B	0.397638	0.638414	0.456628	0.035*	

### Atomic displacement parameters (Å^2^)

**Table:**

	*U*^11^	*U*^22^	*U*^33^	*U*^12^	*U*^13^	*U*^23^
N1	0.0234 (11)	0.0348 (16)	0.0294 (13)	−0.0034 (11)	0.0031 (9)	0.0008 (11)
O1	0.0264 (10)	0.0434 (14)	0.0335 (12)	0.0053 (9)	0.0002 (8)	0.0001 (10)
C1	0.0240 (14)	0.047 (2)	0.0339 (16)	−0.0011 (13)	0.0013 (12)	−0.0078 (15)
C2	0.0194 (13)	0.0348 (18)	0.0365 (17)	−0.0036 (13)	0.0034 (12)	−0.0013 (14)
C3	0.0233 (13)	0.035 (2)	0.047 (2)	0.0023 (13)	0.0047 (13)	−0.0013 (15)
C4	0.0284 (14)	0.033 (2)	0.051 (2)	−0.0017 (13)	0.0120 (14)	−0.0119 (15)
C5	0.0270 (14)	0.0336 (18)	0.0358 (18)	−0.0090 (13)	0.0070 (12)	−0.0041 (14)
C6	0.0412 (17)	0.040 (2)	0.0398 (19)	−0.0157 (16)	0.0151 (14)	−0.0116 (16)
C7	0.0501 (19)	0.048 (2)	0.0286 (17)	−0.0210 (17)	0.0061 (14)	0.0037 (15)
C8	0.0444 (17)	0.043 (2)	0.038 (2)	−0.0089 (16)	−0.0002 (15)	0.0075 (16)
C9	0.0355 (15)	0.034 (2)	0.0377 (19)	−0.0048 (14)	0.0057 (13)	0.0047 (15)
C10	0.0244 (13)	0.0273 (17)	0.0374 (17)	−0.0071 (12)	0.0067 (12)	−0.0008 (13)
C11	0.0232 (12)	0.0280 (17)	0.0322 (16)	−0.0049 (12)	0.0058 (11)	−0.0010 (13)
C12	0.0266 (13)	0.0287 (17)	0.0319 (16)	0.0005 (12)	0.0067 (12)	0.0002 (13)
C13	0.0250 (13)	0.0322 (18)	0.0302 (15)	0.0019 (12)	0.0047 (12)	0.0014 (14)

### Geometric parameters (Å, º)

**Table:**

N1—C1	1.439 (3)	C6—C7	1.369 (5)
N1—C13	1.472 (4)	C6—H6	0.9500
N1—C12	1.470 (4)	C7—C8	1.398 (5)
O1—C2	1.380 (4)	C7—H7	0.9500
O1—C1	1.453 (4)	C8—C9	1.369 (4)
C1—H1A	0.9900	C8—H8	0.9500
C1—H1B	0.9900	C9—C10	1.425 (4)
C2—C11	1.375 (4)	C9—H9	0.9500
C2—C3	1.415 (4)	C10—C11	1.431 (4)
C3—C4	1.365 (5)	C11—C12	1.517 (4)
C3—H3	0.9500	C12—H12A	0.9900
C4—C5	1.418 (4)	C12—H12B	0.9900
C4—H4	0.9500	C13—C13^i^	1.518 (5)
C5—C10	1.415 (4)	C13—H13A	0.9900
C5—C6	1.422 (4)	C13—H13B	0.9900
			
C1—N1—C13	112.9 (2)	C6—C7—H7	120.3
C1—N1—C12	108.6 (2)	C8—C7—H7	120.3
C13—N1—C12	113.4 (2)	C9—C8—C7	121.7 (3)
C2—O1—C1	112.5 (2)	C9—C8—H8	119.2
N1—C1—O1	113.5 (2)	C7—C8—H8	119.2
N1—C1—H1A	108.9	C8—C9—C10	120.4 (3)
O1—C1—H1A	108.9	C8—C9—H9	119.8
N1—C1—H1B	108.9	C10—C9—H9	119.8
O1—C1—H1B	108.9	C5—C10—C9	118.1 (3)
H1A—C1—H1B	107.7	C5—C10—C11	120.0 (3)
C11—C2—O1	122.9 (3)	C9—C10—C11	121.8 (3)
C11—C2—C3	121.9 (3)	C2—C11—C10	118.5 (3)
O1—C2—C3	115.2 (3)	C2—C11—C12	119.8 (3)
C4—C3—C2	119.7 (3)	C10—C11—C12	121.7 (3)
C4—C3—H3	120.1	N1—C12—C11	112.6 (2)
C2—C3—H3	120.1	N1—C12—H12A	109.1
C3—C4—C5	120.8 (3)	C11—C12—H12A	109.1
C3—C4—H4	119.6	N1—C12—H12B	109.1
C5—C4—H4	119.6	C11—C12—H12B	109.1
C10—C5—C4	119.0 (3)	H12A—C12—H12B	107.8
C10—C5—C6	119.5 (3)	N1—C13—C13^i^	110.8 (3)
C4—C5—C6	121.5 (3)	N1—C13—H13A	109.5
C7—C6—C5	120.9 (3)	C13^i^—C13—H13A	109.5
C7—C6—H6	119.5	N1—C13—H13B	109.5
C5—C6—H6	119.5	C13^i^—C13—H13B	109.5
C6—C7—C8	119.4 (3)	H13A—C13—H13B	108.1
			
C13—N1—C1—O1	−62.2 (3)	C6—C5—C10—C11	179.5 (3)
C12—N1—C1—O1	64.5 (3)	C8—C9—C10—C5	−0.3 (4)
C2—O1—C1—N1	−50.6 (3)	C8—C9—C10—C11	−179.0 (3)
C1—O1—C2—C11	16.5 (4)	O1—C2—C11—C10	−179.6 (3)
C1—O1—C2—C3	−164.7 (2)	C3—C2—C11—C10	1.6 (4)
C11—C2—C3—C4	−0.2 (5)	O1—C2—C11—C12	1.3 (4)
O1—C2—C3—C4	−179.0 (3)	C3—C2—C11—C12	−177.5 (3)
C2—C3—C4—C5	−1.5 (5)	C5—C10—C11—C2	−1.4 (4)
C3—C4—C5—C10	1.6 (4)	C9—C10—C11—C2	177.2 (3)
C3—C4—C5—C6	−178.0 (3)	C5—C10—C11—C12	177.6 (3)
C10—C5—C6—C7	−1.0 (5)	C9—C10—C11—C12	−3.7 (4)
C4—C5—C6—C7	178.6 (3)	C1—N1—C12—C11	−43.2 (3)
C5—C6—C7—C8	0.8 (5)	C13—N1—C12—C11	83.2 (3)
C6—C7—C8—C9	−0.3 (5)	C2—C11—C12—N1	12.7 (4)
C7—C8—C9—C10	0.1 (5)	C10—C11—C12—N1	−166.3 (3)
C4—C5—C10—C9	−178.8 (3)	C1—N1—C13—C13^i^	−156.7 (3)
C6—C5—C10—C9	0.8 (4)	C12—N1—C13—C13^i^	79.2 (4)
C4—C5—C10—C11	−0.1 (4)		

Symmetry code: (i) −*x*+1, −*y*+1, −*z*+1.
